# Editorial: Mast Cells: Bridging Host-Microorganism Interactions

**DOI:** 10.3389/fimmu.2022.827375

**Published:** 2022-01-31

**Authors:** Rommel Chacón-Salinas, Anna Di Nardo, Soman N. Abraham

**Affiliations:** ^1^ Departamento de Inmunología, Escuela Nacional de Ciencias Biológicas, Instituto Politécnico Nacional, (ENCB-IPN), Mexico City, Mexico; ^2^ Department of Dermatology, University of California San Diego, San Diego, CA, United States; ^3^ Pathology Department, School of Medicine, Duke University, Durham, NC, United States; ^4^ Department of Immunology, School of Medicine, Duke University, Durham, NC, United States; ^5^ Department of Molecular Genetics and Microbiology, Duke University, Durham, NC, United States

**Keywords:** mast cell (MC), bacteria, virus, fungi, microbiota

Mast cells (MCs) evolved as part of the innate immune system more than 500 million years ago to respond to varying signals from microorganisms that establish different interactions with the host, ranging from mutualistic to parasitic. This Research Topic illustrates how MCs influence the immune system’s relationship with different microorganisms and how these MC responses modulate subsequent host-microorganism interplay.

In a comprehensive overview, Jiménez et al. describe the biology and major functions of MCs with a focus on our current understanding of their interaction with microorganisms, ranging from viruses to parasites. They discuss the importance of MC mechanisms to induce protection against infection and they also illustrate the damage associated with exacerbated MC activation during different infectious diseases.

Yu et al. discuss the importance of MC activation during fungal infections. They review experimental evidence of the role of MCs during infections with the fungi *Candida albicans*, *Aspergillus fumigatus*, and *Sporothrix schenkii*.

Because of their strategic location, MCs are sentinels at epithelial interfaces and are first responders in host-microbiome interactions. MCs primarily interact with microbes *via* innate immune receptors, such as Toll-Like Receptors (TLRs). As Soria-Castro et al. describe, during infection with *Listeria monocytogenes*, MCs responses are mediated through TLR2 activation. However, they can also respond when TLR2 is blocked, using other innate immune receptors.

Draberova et al. describe alternate pathways of MC activation during infection, focusing on Gram-positive bacterial exotoxins that promote cholesterol-dependent cellular lysis. At low concentrations, these exotoxins cause pore formation in the membrane of MCs leading to the activation of cell signaling pathways that induce MC degranulation and production of several inflammatory mediators. Interestingly, other molecules that can modify MC membrane integrity can mimic this activation pathway.

However, the significance of MCs in host defense is more evident when we see MCs also modulate the acquired immune system. Palma et al. illustrate how MCs modulate B-cell responses. Their review describes the critical impacts of MCs on B cells using information from both clinical and laboratory studies. They also discuss the implications of these findings on host responses to infections.

Antibody responses can modulate the responses of MCs to microorganisms, which is illustrated by Mamontov et al. during influenza virus infection, turning the capacity of MCs to recognize viruses into a damaging response. This study elegantly shows that virus-specific non-neutralizing antibodies can interact with MCs upon infection with the avian influenza virus. It is suggested that this antibody-mediated MC response, which includes significant histamine release, could potentially cause side effects when vaccinated subjects are infected with the influenza virus.

A second level of MC interactions with the adaptive immune response is evident during T cell activation following viral infections. Employing MC deficient mice, Hackler et al. reveal the critical role played by MCs in promoting adequate dendritic cell activation and the induction of specific CD8+ T-cell responses that limit lymphocytic choriomeningitis virus infections.

The ability of MCs to participate in the control of bacterial infections can be exploited by employing distinct polypeptides that target specific molecules on MCs. For example, Amponnawarat et al. show that murepavadin, a lipopolysaccharide transport protein D (LptD)- binding host defense peptidomimetic antimicrobial peptide that targets multi-drug resistant *Pseudomonas aureginosa*, can activate MCs *in vitro* through Mas-Related G Protein-Coupled Receptor member X2 (MRGPRX2), inducing degranulation and cytokine production. Moreover, *in vivo*, they revealed that murepavadin increases vascular permeability in mice.

Finally, Yeh et al. reveal that MC interactions with microorganisms can be a bidirectional response, where interactions with microbiota impact MC distribution in the lungs. They show that mice captured in the wild exhibit high numbers of MCs in the lung parenchyma compared to conventional pathogen-free laboratory animals. Remarkably, when laboratory mice are bred in conditions that mimic the environment in the wild, they exhibit increased numbers of MCs in the lungs.

In conclusion, this Research Topic updates the current state of knowledge regarding MC crosstalk with mutualistic and parasitic microorganisms and how this interaction impacts innate and adaptive immune response modulating host homeostasis. The role of MCs clearly depends on the nature of its interactions with specific microorganisms, as these interactions can promote either protection or damage to the host. Purposeful modulation of some of these interactions could represent new therapeutic targets and novel strategies to combat infectious diseases.

## Author Contributions

All authors made a substantial, direct, and intellectual contribution to this work and approved it for publication.

## Funding

RC-S was supported by Secretaría de Investigación y Posgrado I.P.N, (SIP-IPN).

## Conflict of Interest

The authors declare that the research was conducted in the absence of any commercial or financial relationships that could be construed as a potential conflict of interest.

## Publisher’s Note

All claims expressed in this article are solely those of the authors and do not necessarily represent those of their affiliated organizations, or those of the publisher, the editors and the reviewers. Any product that may be evaluated in this article, or claim that may be made by its manufacturer, is not guaranteed or endorsed by the publisher.

